# The Adenosine Hypothesis Revisited: Modulation of Coupling between Myocardial Perfusion and Arterial Compliance

**DOI:** 10.3389/fphys.2017.00824

**Published:** 2017-10-20

**Authors:** Geoffrey P. Dobson, Aryadi Arsyad, Hayley L. Letson

**Affiliations:** ^1^Heart, Trauma and Sepsis Research Laboratory, College of Medicine and Dentistry, James Cook University, Townsville, QLD, Australia; ^2^Physiology Department, Medical Faculty, Hasanuddin University, Makassar, Indonesia

**Keywords:** rat aorta, adenosine, relaxation, ventricular-arterial coupling, vasodilation, compliance

## Abstract

For over four decades the thoracic aortic ring model has become one of the most widely used methods to study vascular reactivity and electromechanical coupling. A question that is rarely asked, however, is what function does a drug-mediated relaxation (or contraction) in this model serve in the intact system? The physiological significance of adenosine relaxation in rings isolated from large elastic conduit arteries from a wide range of species remains largely unknown. We propose that adenosine relaxation increases aortic compliance in acute stress states and facilitates ventricular-arterial (VA) coupling, and thereby links compliance and coronary artery perfusion to myocardial energy metabolism. In 1963 Berne argued that adenosine acts as a local negative feedback regulator between oxygen supply and demand in the heart during hypoxic/ischemic stress. The adenosine VA coupling hypothesis extends and enhances Berne's “adenosine hypothesis” from a local regulatory scheme in the heart to include conduit arterial function. In multicellular organisms, evolution may have selected adenosine, nitric oxide, and other vascular mediators, to modulate VA coupling for optimal transfer of oxygen (and nutrients) from the lung, heart, large conduit arteries, arterioles and capillaries to respiring mitochondria. Finally, a discussion of the potential clinical significance of adenosine modulation of VA coupling is extended to vascular aging and disease, including hypertension, diabetes, obesity, coronary artery disease and heart failure.

From these studies emerged the idea that a labile substance was released from the heart (presumably from the myocardial cells) when the oxygen supply became inadequate for the oxygen needs of the heart, and the labile substance dilated the coronary-resistance vessels, thereby increasing coronary blood flow and restoring the balance between oxygen need and supply.

Robert M. Berne (originator of the “Adenosine Hypothesis”). Quoted from (Berne, 1998), p14

## Background

Berne's “labile substance” was adenosine, and the same “adenine compound” was shown to influence heart function some 30 years earlier by Drury and Szent-Gyorgyi (1929). Today, we know adenosine as a naturally occurring, multi-functional, endogenous purine nucleoside that plays a key role in the cardiovascular system by activating adenosine receptor subtypes on constituent cardiac and vascular cells: A_1_, A_2A_, A_2B_, and A_3_ (Vinten-Johansen et al., 1999; Fredholm et al., 2001; Linden, 2001; Jacobson and Gao, 2006; Leal et al., 2008; Headrick et al., 2013). Adenosine also regulates cardiovascular function indirectly from its effects on central and peripheral nervous systems (Minic et al., 2015). Adenosine is produced by most cells in the body in response to metabolic stress or injury and also assists in the modulation of the early inflammatory and innate immune responses (Hasko and Cronstein, 2004; Headrick et al., 2013). Adenosine has been shown to inhibit the release of proinflammatory cytokines TNF-α, IL-6, and IL-12, and stimulate the release of anti-inflammatory cytokines IL-8, IL-10, and VEGF by macrophages, and there is accumulating evidence that it promotes angiogenesis, tissue remodeling and wound healing (Hasko and Cronstein, 2004; Chan and Cronstein, 2010; Ernens et al., 2015; Vecchio et al., 2017). The focus of this perspective is on a new role for adenosine as a possible “aortic compliance regulator” that links myocardial perfusion to arterial compliance in the cardiovascular system.

## The adenosine hypothesis: metabolic control of coronary blood flow

**Figure 1:**
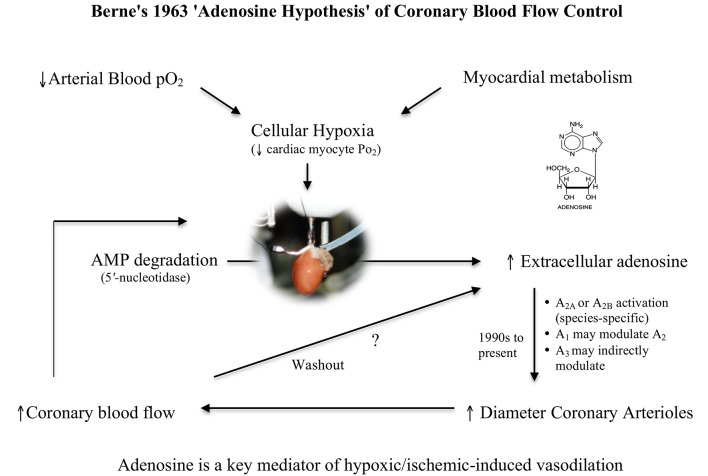
Schematic of Berne's “adenosine hypothesis,” which proposes that myocardial hypoxia leads to the breakdown of adenine nucleotides and formation of adenosine, which “diffuses out of the cell and reaches the coronary arterioles via the interstitial fluid and produces arteriolar dilation” (Berne, 1963). As blood flow increases and metabolic energy demand is met, adenine nucleotides decrease leading to decreases in interstitial adenosine “by washout and enzymatic destruction” (Berne, 1963). In this way, adenosine serves as a local negative feedback regulator of oxygen supply and oxygen demand in the heart.

Since Berne first proposed the “adenosine hypothesis” over 50 years ago (Berne, 1963, 1980), there has been much controversy about its wider importance to the regulation of coronary flow in heart, and other organs in normal and pathological states (Collis, 1989; Feliciano and Henning, 1999; Feigl, 2004). Berne originally proposed that during hypoxia (or ischemia) adenosine was a potent local negative feedback regulator between myocardial oxygen supply and oxygen demand. As myocardial pO_2_ decreases during hypoxia (i.e., increased oxygen demand), adenosine is formed in the myocyte from the breakdown of adenosine 5′ monophosphate (AMP), diffuses across the interstitial space to the coronary artery and causes vasodilation (i.e., increased oxygen supply) (Figure 1). Berne's hypothesis was bold and far-reaching because during the 1960s there was little or no knowledge on the mechanisms of how adenosine dilated the vessel wall.

In the late 1970s the “adenosine hypothesis” entered a new era when adenosine receptors were identified and functionally characterized (Fredholm et al., 2001; Linden, 2001; Jacobson and Gao, 2006). A decade later Headrick and Berne showed that adenosine dilation of guinea-pig aorta involved activation of the adenosine A_2_ receptor subtype, with 30% relaxation being derived from the endothelium and 70% from underlying smooth muscle (Headrick and Berne, 1990). Today, coronary vasodilation involves principally the activation of A_2A_ or A_2B_ receptors depending upon animal species, and a complex crosstalk between A_1_ and A_3_ receptor subtypes that remain poorly defined (Headrick et al., 2013). Notwithstanding the advances in the 1990s, in a memoir written by Berne at the turn of the century, he jokingly wrote about the ongoing controversies surrounding his “adenosine hypothesis”:

“But the worst blow was when I was skiing with Larry Rowell and Eric Feigl and some of their postdocs on one of my many visits to Seattle. As I lost control on a steep slope and proceeded to crash, a voice rang out from one of the postdocs on the ski-lift as he viewed the carnage … There goes the adenosine hypothesis.”

(Berne, 1998, p. 14).

Since that time increasing support has accrued for the concept that adenosine acts as a “negative feedback regulator” in most tissues during hypoxia/ischemia, including skeletal muscle and brain (Feigl, 2004). In addition, adenosine has many other important cellular protective properties following hypoxia/ischemia or injury (see Background above). More recently, adenosine has been identified as one of the self-preservation signals of ischemic preconditioning and post-conditioning in the heart and most organs of the body, which appears to involve a memory-like function leading to the activation of multiple survival kinase pathways (Vinten-Johansen et al., 2007; Headrick et al., 2013). Notwithstanding the evolutionary importance of adenosine to flow regulation and energy metabolism, it must be acknowledged that adenosine is only one of many vascular modulators that link blood flow supply to metabolic demand. Other modulators include membrane potential, nitric oxide (NO), K_ATP_ channel openers, catecholamines, endothelins, prostanoids, opioids, reactive oxygen species, pO_2_, pCO_2_, and pH (Furchgott, 1983; Wilkinson et al., 2002; Duncker and Bache, 2008; Bellien et al., 2010; Marti et al., 2012; Quillon et al., 2015; Félétou, 2016; Behringer, 2017).

## Physiological functions of the large elastic aorta

A large number of experimental studies spanning over three decades have demonstrated that adenosine is a potent vasorelaxant of thoracic aortic rings from a wide range of species (Heistad et al., 1978; Lewis and Hourani, 1997; Ray and Marshall, 2006; Mustafa et al., 2009; Ponnoth et al., 2009; Headrick et al., 2013; Arsyad and Dobson, 2016). An important question that is rarely asked, however, is how does relaxation of an isolated aortic ring translate into function in the intact system? And why has adenosine signaling been selected and highly conserved in the thoracic aorta? To help answer these questions, we will first consider the functional differences between the aorta and smaller muscular arterioles. The aorta operates as a biological “windkessel” or buffering reservoir that stores a portion (~10%) of kinetic energy from stroke work during systole, and transfers it during diastole to maintain a relative constant pressure and flow to the periphery (London, 2005; Erbel and Eggebrecht, 2006; Steppan et al., 2011). Thus the aorta is not a resistance vessel constantly dilating and relaxing, like the smaller muscular coronary arteries or peripheral arterioles, to meet the oxygen demands of a tissue. The aorta and other conduit arteries (carotid, iliac, pulmonary trunk and brachiocephalic trunk), are highly elastic vessels that *dampen the pulsatile flow generated by heart contractions to ensure an almost continuous flow of oxygenated blood to the periphery* (AlGhatrif and Lakatta, 2015).

Elastic arteries are therefore highly compliant, a property not associated with smaller muscular arterioles (Marti et al., 2012). Compliance is defined as the ability of an artery to expand in response to pressure changes (Table 1). The degree of elasticity is contingent upon three main factors: (1) differences in elastin-to-collagen fiber ratios along the vessel, (2) differences in endothelium-smooth muscle responses to cell stretch as a result of stroke work, and (3) differences in sympathetic nerve innervation affecting wall properties (Ooi et al., 2008; Steppan et al., 2011; Marti et al., 2012; Zhipeng et al., 2014). Put simply, compliance is the ability of a vessel to stretch, hold volume and release it during the cardiac cycle. It is directly related to distensibility and inversely related to stiffness (Table 1). The more distal muscular arteries, such as the femoral artery, are less distensible from having a lower elastin-to-collagen ratio compared to the aorta (Zieman et al., 2005; Quinn et al., 2012). Interestingly, arterial compliance and distensibility are inversely related to heart rate, with a higher heart rate having a stiffening effect on elastic arteries, and little effect on muscular arteries (Mangoni et al., 1996). In addition, compliance is known to influence coronary blood flow, an idea first proposed by Bouvrain and Levy (1981).

**Table 1:** Definitions of key terms and methodologies.

**Parameter**	**Definition**	**Physiological significance**
Compliance (C) (ml × mmHg^−1^)	$\text{C}=\frac{\Delta \text{Blood Volume}}{\Delta \text{Blood Pressure}}$	An “index of elasticity” of large conduit arteries. Compliance is directly related to “distensibility,” and inversely related to stiffness and elastance (see Ea below). The endothelium because of its capacity to modulate smooth muscle tone modulates compliance
Ventricular-Arterial (VA) Coupling	$\text{VA}=\frac{\text{Arterial Elastance}}{\text{LV Elastance}}$ where LV = Left ventricular	A measure of mechanical efficiency of the cardiovascular system from assessing the interactions between heart performance and vascular function. When Ea/Ees = 1.0, the efficiency of the system is optimal meaning that the left ventricle is providing sufficient SV at its lowest possible myocardial energy consumption. When Ea/Ees < 1.0 (hypoxia, ischemia, shock, sepsis, traumatic brain injury) efficiency is decreased
Arterial Elastance (Ea)	$\text{Ea }=\frac{\text{ESP}}{\text{SV}},\text{ mmHg}/\text{ml}$ ESP = LV end-systolic pressure = [2 × (systolic BP + diastolic BP)]/3 where BP is blood pressure or ESP = 0.9 × systolic BP SV, stroke volume 0.9 is a factor that accounts for ESP occurring slightly after peak systolic BP	An arterial index that estimates the capability of the arterial vessels to increase pressure when stroke volume increases. Ea is a measure of the total arterial afterload on the heart including arterial wall stiffness, compliance and outflow vascular resistance, and systolic and diastolic time intervals. Thus, Ea lumps the steady and pulsatile components of the arterial load into a single number
LV elastance (Ees)	$\text{Ees }=\frac{\text{ESP}}{\text{ESV}}-\text{Vo},\text{ mmHg}/\text{ml}$ ESP (see Ea above) ESV is LV end-systolic volume Vo is x-axis volume intercept of the end-systolic P-V relationship	Ees is a load-independent index of LV contractility. Index also takes into account stiffness, compliance, fibrosis, contraction synchrony and geometric LV chamber dimensions. Ees is an integrated measure of LV systolic performance to pump blood into the arterial tree and does not change substantially with changes in heart rate
Pulse wave velocity (PWV)	Propagation speed of the wave along the large arteries	PWV is inversely related with BP, and is higher as arteries become stiffer
Invasive “Direct” method	Left ventricular pressure-volume (PV) loops. Ea (see above). Ees is the slope of the end-systolic PV relationship. End-systolic PV relationship assumes independent of load, and that slope is linear	Suga et al., 1993; Chantler et al., 2008; Guarracino et al., 2013
Non-invasive Method	Echocardiographic assessment of LV end-diastolic and end-systolic areas, and blood pressures. Ea (see right). A single beat measure of Ees is calculated from ESP/ESV and assumes Vo is zero	The non-invasive ESP method for Ea or Ees accurately predicts LV PV loop measurements of ESP, as does the ESP/ESV ratio Chen et al., 2001; Najjar et al., 2004; Chantler et al., 2008; Guarracino et al., 2013.

## The adenosine ventricular-arterial (VA) coupling hypothesis

**Figure 2:**
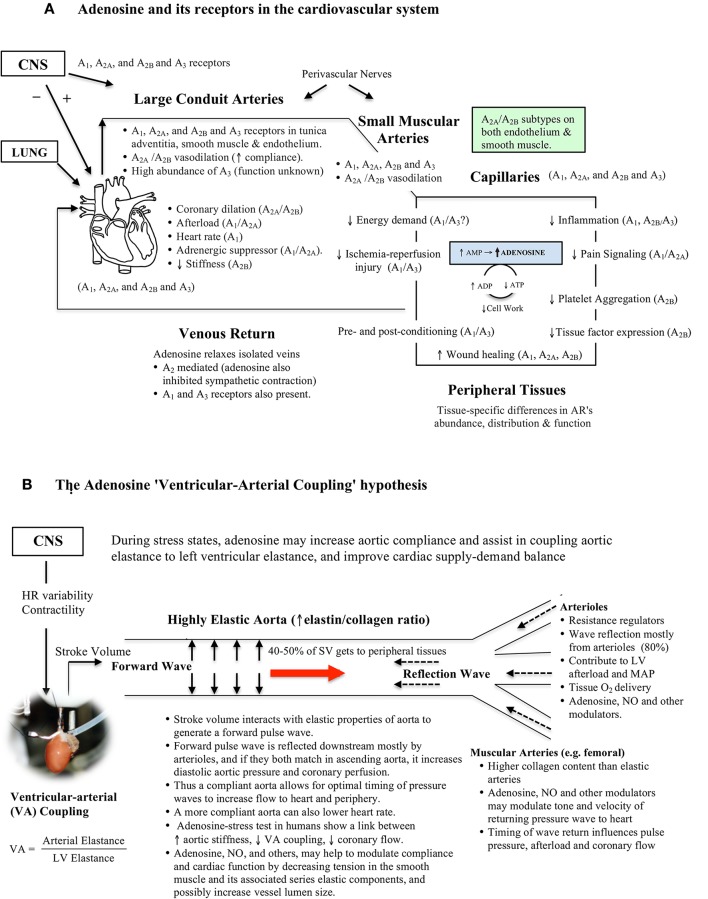
**(A)** Schematic of adenosine's ubiquitous distribution in the cardiovascular system. Adenosine receptors are coupled to G-proteins with diverse functions. Adenosine is formed and released from most active cells when they are metabolically stressed. In the rat thoracic aorta all adenosine receptor subtypes are located in the three layers of the vessel wall. The physiological significance of adenosine relaxation in large conduit arteries remains largely unknown. Data was obtained from the literature (Fredholm et al., 2001; Tabrizchi and Bedi, 2001; Jacobson and Gao, 2006; Leal et al., 2008; Headrick et al., 2013; Minic et al., 2015). **(B)** The adenosine Ventricular-Arterial (VA) coupling hypothesis proposes a link between adenosine, arterial compliance, stiffness and beat-to-beat coupling of cardiac systolic and diastolic function during times of acute stress. Adenosine may increase compliance by decreasing tension in smooth muscle, and its associated series elastic elements. When the heart ejects blood at a rate and volume that matches the capability of the arterial system to receive it, both cardiovascular performance and cardiac energetics are believed to be optimal (VA coupling ratio = 1.0) (see Table 1).

In contrast to the relationship between myocardial adenosine production and coronary artery perfusion, we propose a different role for adenosine in the thoracic aorta. We propose that adenosine may facilitate coupling between left ventricular output and the ability of the arterial system to receive the blood by modulating aortic compliance (Figures 2A,B). This transfer function is termed ventricular-arterial (VA) coupling, and is the ratio of arterial elastance to left-ventricular elastance (Table 1) (Suga et al., 1993; Chen et al., 2001; Najjar et al., 2004; Chantler et al., 2008; Guarracino et al., 2013). VA coupling has consistently been shown to be a reliable and effective index of cardiovascular performance to maintain oxygen supply to respiring mitochondria (Najjar et al., 2004; Chantler et al., 2008; Guarracino et al., 2013). When this coupling ratio is close to unity, the efficiency of the system is considered to be optimal, and during acute and chronic altered hemodynamic states, such as low cardiac output, the ratio becomes less than one (Table 1). The VA coupling ratio also reflects cardiac energetics, and the balance between myocardial oxygen consumption and mechanical energy required to perform cardiac work (stroke work times heart rate) to propel blood forward to the periphery (Chen et al., 2001; Najjar et al., 2004; Chantler et al., 2008; Guarracino et al., 2013).

This new “compliance” role for adenosine may apply to both beat-to-beat cycling during normal cardiac work transitions and acute stress conditions such as during ischemia, stroke, hemorrhagic shock, and other trauma states (Figures 2A,B). In a porcine model of hemorrhagic shock, Jonker and colleagues reported that the thoracic aorta decreases in diameter by 40% and pulsatility decreases by 62% during blood loss compared to baseline (Jonker et al., 2011).

In the thoracic aorta, adenosine may operate upstream of other compliance regulators, such as NO (Ray and Marshall, 2006; Quillon et al., 2015). Endothelium-derived NO has been shown to regulate the elastic properties of conduit arteries, and wave profiles, by increasing vascular smooth muscle relaxation (Wilkinson et al., 2002; Bellien et al., 2010; Marti et al., 2012). We recently extended these studies and showed that adenosine's ability to dilate isolated rat thoracic aortic rings involved endothelial NO and a complex interplay between smooth muscle A_2a_ subtype and voltage-dependent K_v_, SarcK_ATP_, and MitoK_ATP_ channels (Arsyad and Dobson, 2016). A possible upstream scenario in the thoracic aorta is that adenosine may bind to one or more endothelial G-protein coupled receptors (e.g., A_2A_), increase cytosolic Ca^2+^, activate endothelial NO synthase and thereby produce NO. At the same time, increased cytosolic Ca^2+^ may activate small- and intermediate-conductance activated K^+^ channels to produce endothelium-dependent membrane hyperpolarization (EDH) (Félétou and Vanhoutte, 2007; Shimokawa and Godo, 2016; Behringer, 2017). Adenosine, NO and EDH may work in concert to relax the underlying smooth muscle and increase arterial compliance.

The cardiovascular significance of vasoactive drugs changing smooth muscle tone of the elastic aorta was recognized by Bank (1997) when he wrote:

“Vasoactive drugs alter smooth muscle tone not only in arterial resistance vessels, but also in large conduit arteries … The resultant changes in smooth muscle tone alter both conduit vessel size and stiffness and hence influence pulsatile components of left ventricular afterload” (Bank, 1997).

Bank further reported that the effect to nitroglycerin, an NO donor commonly used to treat myocardial ischemia in humans by increasing coronary blood flow, also decreased pulse wave velocity and increased arterial compliance from its dilation properties and effects on heart chamber geometry (Bank and Kaiser, 1998). In multicellular organisms, evolution may have selected adenosine, nitric oxide and other modulators, to provide optimal oxygen and nutrient transfer along vascular branching networks from the lung, heart, large arteries and arterioles, capillaries to respiring mitochondria (Dobson, 2003).

Additional support for the adenosine VA coupling hypothesis comes from adenosine's widespread distribution throughout the cardiovascular system, and long recognized linkage between transitioning from aerobic to anaerobic metabolism, AMP production and adenosine formation (see Figure 2A). Since adenosine has a very short half-life (secs), its ability to be rapidly activate and inactivate may constitute a high-gain feedback control system between the heart and conduit arteries. Adenosine levels have been shown to change in heart muscle during a single cardiac cycle, with higher levels being produced during systole than diastole, indicating rapid metabolism of adenosine (Thompson et al., 1980). In addition, adenosine depresses heart pacemaker activity (negative chronotropic effect), decreases atrial and ventricle contractility (negative inotropic effect), depresses cardiac automaticity (dromotropic effect), and decreases heart diastolic stiffness (lusitropic effect) (Figure 2A) (Vinten-Johansen et al., 1999; Jacobson and Gao, 2006; Mustafa et al., 2009; Headrick et al., 2013; Minic et al., 2015).

Perhaps the most compelling set of human data supporting a role for adenosine in VA coupling comes from Wong and colleagues who reported a relationship between increased aortic stiffness, lower VA coupling and lower coronary blood flow in patients with stable angina undergoing an adenosine stress test (Wong et al., 2010). Unfortunately, these authors did not comment on a possible adenosine spatial-temporal link between arterial stiffness and coronary perfusion, presumably because it was a clinical diagnostic test. Leung and colleagues also showed in humans that aortic stiffness affected coronary blood flow during percutaneous coronary intervention (PCI), and that a more compliant aorta was associated with a greater improvement in adenosine-induced increases in coronary blood flow compared to patients which had a stiffer aorta (Leung et al., 2006). Pagliaro and colleagues also showed in dogs that a combination of low dose adenosine and enhanced perfusion pulsatility markedly increased coronary blood flow by mechanisms involving adenosine receptors, NO and K_ATP_ channels, and possibly other purinergic receptors (Pagliaro et al., 1999; Kass, 2005; Burnstock and Ralevic, 2013). Adenosine has also been reported to dilate the vasa vasorum that supplies blood to the aortic media (Heistad et al., 1978), which may also influence compliance. Future studies are required to extend these clinical and experimental observations, and examine if the effects of adenosine (and NO) in large elastic arteries change in concentration under different cardiac workload conditions. If changes are found they may reflect differential activation of adenosine receptor subtypes (A_1_, A_2A_, A_2B_, and A_3_) (Figure 2A). Interestingly, Leal and colleagues reported that the rat abdominal aorta had 35-times higher abundance of the A_3_ receptor subtype than in a muscular tail artery, and a significantly lower abundance of A_1_ and A_2A_ receptors in the conduit (Leal et al., 2008), which may imply different functional roles (Figures 2A,B).

Finally, since the heart and elastic and geometric properties of conduit arteries are important predictors of aging and disease progression, the adenosine VA hypothesis may offer a new conceptual scheme for future research and therapeutic intervention. Questions include whether adenosine levels change in large conduit arteries and along their length in healthy vs. older patients with hypertension, diabetes mellitus, obesity, coronary artery disease and/or heart failure (AlGhatrif and Lakatta, 2015; Yurdagul et al., 2016). Further, do these changes correlate with changes in myocardial perfusion, VA coupling and wall stiffness? Indeed, progressive vessel wall stiffening, and microcirculatory endothelial dysfunction has been reported along the wall of the thoracic aorta, which precedes hypertension, diabetes and cardiovascular diseases, including aortic aneurysms (Erbel and Eggebrecht, 2006; Huveneers et al., 2015). Recently the VA coupling ratio was found to be profoundly decreased in a population of centenarians without overt cardiovascular disease, particularly in women (Sonaglioni et al., 2017), highlighting again the importance of understanding the changes between heart, aortic compliance and vascular aging. In conclusion, we propose that adenosine may play an important role in maintaining aortic wall integrity and compliance for optimal VA coupling, coronary perfusion and downstream regulation of tissue blood flow and oxygenation. This hypothesis warrants further testing.

## Author contributions

All authors listed, have made substantial, direct and intellectual contribution to the work, and approved it for publication.

### Conflict of interest statement

The authors declare that the research was conducted in the absence of any commercial or financial relationships that could be construed as a potential conflict of interest.
